# Rhamnosidase activity of selected probiotics and their ability to hydrolyse flavonoid rhamnoglucosides

**DOI:** 10.1007/s00449-017-1860-5

**Published:** 2017-11-10

**Authors:** Monika Mueller, Barbara Zartl, Agnes Schleritzko, Margit Stenzl, Helmut Viernstein, Frank M. Unger

**Affiliations:** Department of Pharmaceutical Technology and Biopharmaceutics, University of Vienna, Althanstrasse 14, 1090 Vienna, Austria

**Keywords:** Rhamnosidase, Hesperidin, Naringin, Rutin, Narcissin, Probiotics

## Abstract

Bioavailability of flavonoids is low, especially when occurring as rhamnoglucosides. Thus, the hydrolysis of rutin, hesperidin, naringin and a mixture of narcissin and rutin (from *Cyrtosperma johnstonii*) by 14 selected probiotics was tested. All strains showed rhamnosidase activity as shown using 4-nitrophenyl α-l-rhamnopyranoside as a substrate. Hesperidin was hydrolysed by 8–27% after 4 and up to 80% after 10 days and narcissin to 14–56% after 4 and 25–97% after 10 days. Rutin was hardly hydrolysed with a conversion rate ranging from 0 to 5% after 10 days. In the presence of narcissin, the hydrolysis of rutin was increased indicating that narcissin acts as an inducer. The rhamnosidase activity as well as the ability to hydrolyse flavonoid rhamnoglucosides was highly strain specific. Naringin was not hydrolysed by rhamnosidase from probiotics, not even by the purified recombinant enzyme, only by fungal rhamnosidase. In conclusion, rhamnosidases from the tested probiotics are substrate specific cleaving hesperidin, narcissin and to a small extent rutin, but not naringin.

**Electronic supplementary material:**

The online version of this article (10.1007/s00449-017-1860-5) contains supplementary material, which is available to authorized users.

## Introduction

As secondary plant metabolites with important contents in human diets [1, 2], flavonoids occur in a wide variety of compounds comprising six subclasses [3, 4]. Flavonols, the most common flavonoids in foods include quercetin, its glycoside rutin (quercetin-3-rutinoside), kaempferol, isorhamnetin and its glycoside narcissin (isorhamnetin-3-rutinoside) [5, 6]. Main sources of these compounds are onions and broccoli, but also red wine and tea. Flavanones with their important representatives naringenin (grapefruits) and hesperetin (oranges), and their glycosides, are also found in other human foods such as tomatoes and aromatic plants [4]. The most familiar flavones in diet are apigenin and luteolin which occur in red pepper [5] and celery [5, 7]; isoflavones are present in soybean-derived products or red clover and show commonalities with estrogens [8] and flavanols occur in apricots, red wine, green tea and chocolate [4].

Interest in flavonoids has been growing because of their biological properties such as anti-inflammatory, antioxidant, antimutagenic, antiproliferative and antiatherogenic effects as shown for quercetin, isorhamnetin, naringenin, hesperetin and its glycosides rutin, narcissin, naringin (naringenin-7-neohesperidoside) and hesperidin (hesperetin-7-rutinoside) [9–12]. Thus, those compounds play a role in amelioration of various diseases or disorders with high prevalence globally including neurodegenerative diseases, osteoporosis, cancer or cardiovascular diseases including atherosclerosis, thrombosis and hypertension.

Most flavonoids are found in glycosylated form, often glycosylated by rutinose (6-*O*-α-l-rhamnopyranosyl-d-glucose), as rutinosides (e.g. rutin, hesperidin, narcissin) or by neohesperidose (2-*O*-α-l-rhamnopyranosyl-d-glucose) as neohesperidosides (e.g. naringin). Glycosylation by rutinose or neohesperidose hinders the absorption of the flavonoids in the small intestine [6]. Due to a lack of human intestinal α-l-rhamnosidase, a prior enzymatic hydrolysis increases bioavailability and bioactivity of flavonoid glycosides [13–15]. α-l-Rhamnosidase [EC 3.2.1.40] cleaves terminal α-l-rhamnose from several natural products [16] and is applied for de-bittering of fruit juices [17] or enhancing wine aroma [18]. Beside its biotechnological applications, there is an upcoming interest in α-l-rhamnosidase for enhancing the bioavailability of natural glycosides such as flavonoids rhamnoglucosides by hydrolysing the l-rhamnose [19]. Due to their wide availability, high selectivity, low cost and fast reaction behaviour, the use of enzymes to modify the structure and to improve the physicochemical and biological properties of flavonoids has become an interesting alternative [15]. Enzymes from probiotics have the advantage of safety and possible combined health effects of polyphenols and probiotics [20].

Here, we tested the rhamnosidase activity of 14 selected probiotic strains and their ability to hydrolyse naringin, hesperidin, rutin and the main constituents (rutin and narcissin) of an ethanolic extract of *Cyrtosperma johnstonii* which exerts antioxidant activity and cytotoxicity towards cancer cells as previously shown [21].

## Materials and methods

### Chemicals, plant extract and bacterial strains

Acetonitrile was purchased from Promochem (Wesel, Germany), recombinant α-l-rhamnosidase from prokaryotic source (190 U/mg) was obtained in purified form from Megazyme (Wicklow, Ireland), β-glycosidase/α-l-rhamnosidase mixture [Cellulase from (*A*) niger, 0.45 U/mg] from Sigma–Aldrich (St. Louis, MO, USA), flavonoid glycosides, aglycones and all other chemicals from Sigma–Aldrich [or Merck (Darmstadt, Germany)]. Dried powder of *C. johnstonii* rhizomes (voucher specimen No 010112, gift of Chiang Mai University, Thailand) was extracted with ethanol for 24 h, filtrated and evaporated to obtain a dried crude extract. The probiotic strains used in this study included *Lactobacillus* (*L*.) *paracasei* ssp. *paracasei* CRL 431 (ATCC 55544), *L. paracasei* ssp. *paracasei* (DN114001), *L. paracasei* ssp. *paracasei* DSM 20312 (Shirota), *L. rhamnosus* GG (ATCC 53103), *L. reuteri* (ATCC 55730), *L. acidophilus* LA-5 (DSM 13241), *Bifidobacterium* (*B*.) *animalis* ssp. *lactis BB12* (DSM 15954), (*B*) *longum* ssp. *infantis, L. plantarum* (ATCC 15697), *L. brevis* (ATCC 367), *L*. *delbrueckii* ssp. *bulgaricus* (DSM 20081), *Lactococcus* (*Lc*.) *lactis* ssp. *lactis* (SR 3.54; NCIMB 30117), *L. fermentum, Streptococcus* (*S*.) *salivarius* ssp. *thermophilus* (ATCC 19258). Man–Rogosa–Sharpe (MRS) liquid medium was prepared as described previously [22].

### Hydrolysis of the flavonoid rhamnoglucosides using rhamnosidase

As a positive control, glycosides (10^−4^ M) were incubated with a recombinant α-l-rhamnosidase (1 U/mL) or an enzyme mixture from *A. niger* (5 U/mL β-glucosidase with rhamnosidase side activity) in 100 mM sodium phosphate buffer pH = 6.5 at 37 °C for up to 3 h. The negative control was incubated without enzyme; 4-nitrophenyl α-l-rhamnopyranoside (NPRP) was used as reference substance. Samples were drawn at certain time points, diluted with methanol (1:1) to precipitate the enzymes and clarified using centrifugation at 13,000 rpm and 4 °C for 15 min. Supernatants were analysed using high performance liquid chromatography (HPLC). All experiments were performed in independent triplicates.

### Analysis of the hydrolysis of flavonol rutinosides by probiotics

Probiotics were cultivated overnight in MRS medium with glucose under anaerobic conditions at 37 °C, diluted with MRS medium without carbohydrates to an OD_600_ of 0.2 and further incubated for 48 h. Subsequently, the bacterial suspension was diluted to an OD_600_ of 0.2 in MRS medium without carbohydrate source, supplemented with 10^−4^ M NPRP, nitrophenylβ-d-glucopyranoside (NPGP), rutin, naringin, hesperidin or with extract of *C. johnstonii* (main compounds ~ 10^−4^ M) and incubated at 37 °C for up to 10 days under anaerobic conditions. Samples of NPRP were drawn after 0, 1, 2, 3, 4, 7 days and samples of the rutinosides after 0, 4, 7 and 10 days. The negative control was incubated without bacteria for 10 days. Samples were diluted with methanol (1:1) and clarified using centrifugation at 13,000 rpm and 4 °C for 15 min. Supernatants were analysed using HPLC. All experiments were performed in triplicates.

### Analysis of flavonols and their glycosides by HPLC

The extent of hydrolysis was determined using HPLC with ultraviolet detection (HPLC-UV) on an UltiMate3000 HPLC system (Thermo Fisher Scientific, Waltham, MA, USA) connected to a security guard cartridge followed by a Kinetex C-18 column (5 µm C18, 4.6 × 150 mm, Phenomenex, Torrance, CA, USA). The mobile phase consisted of solvent A (water–acetonitrile 95:5, 0.1% trifluoroacetic acid, TFA) and solvent B (acetonitrile, 0.1% TFA). A linear gradient from 0 to 100% B was applied with a flow rate of 0.5 mL/min: from 0 to 2.5 min B was increased to 15%; from 2.5 to 7.5 min B was increased to 20%, until 13.5 min B was increased to 25%; until 14 min B was increased to 30%; until 15 min to 35%; until 17 min to 50%; until 22 min to 100%; after 2 min at 100%, the column was equilibrated with 100% A. The elution profile was recorded using a photodiode array detector PDA-100 set at the respective absorption maxima of the compounds (305 nm for NPRP, NPGP and nitrophenol; 280 nm for naringenin, hesperetin and their glycosides, and 350 nm for quercetin, isorhamnetin and their glycosides). The respective retention times were 11.8 min for NPRP, 7.7 min for NPGP and 12.8 for nitrophenol; 12.9 for rutin, 13.6 for isoquercitrin and 22.1 for quercetin; 14.6 for naringin and 23.8 for naringenin; 15.8 for hesperidin and 24.3 for hesperetin; 15.7 for isorhamnetinrutinosid, 16.6 for isorhamnetin-3-*O*-glucoside and 24.6 for isorhamnetin. Flavonoids and their glycosides were quantified using Chromeleon software. The results are shown as mean (from a triplicate) ± standard deviation.

### Analysis of rhamnosidase by SDS–PAGE

The rhamnosidase expression was detected in the cell lysates of *L. acidophilus* as an example and the molecular weight was compared to the recombinant rhamnosidase and the enzyme mixture from *A. niger* using sodium dodecyl sulphate polyacrylamide gel electrophoresis (SDS–PAGE). Bacterial culture samples were taken at different time points and centrifuged at 12,500 rpm and RT for 10 min. The pellets were re-suspended in diluted cell lysis reagent (CelLytic™ B lysis reagent, Sigma Aldrich) and incubated 10 min at RT under mixing at 350 rpm. Samples were centrifuged (12,500 rpm, 10 min, RT) and the supernatants were diluted with Laemmli sample buffer (BIO-RAD). Samples were incubated at 99 °C for 10 min, mixed at 350 rpm and afterward loaded to the gel (12% Mini-PROTEAN^®^ TGX™ precast protein gels, 15-well, 15 µL, BIO-RAD) and run with electrophoreses buffer (10× Tris/Glycine/SDS, BIO-RAD) at 130 V. As protein marker peqGOLD protein marker IV (10–170 kDa, VWR) was used. Proteins were stained with Coomassie brilliant blue R-250.

## Results

### Hydrolysis of flavonoid rhamnoglucosides using purified rhamnosidases

The recombinant rhamnosidase (used as positive control) was highly efficient for the hydrolysis of the flavonoid glycosides and NPRP (Table 1). 50% of rutin were hydrolysed into isoquercitrin after 20 min and 100% after 130 min at an incubation temperature of 37 °C. Naringin was not hydrolysed. Hesperidin showed a lower hydrolysis rate than rutin with 50% of conversion to hesperetin-7-*O*-glucoside after 90 min, whereas a 100% hydrolysis was not reached. No hydrolysis was observed in the negative controls without enzymes for up to 72 h. The hydrolysis of all tested flavonoid glycosides was higher at 50 °C.

**Table 1:** Hydrolysis of rhamnose from NPRP, rutin, naringin and hesperidin by rhamnosidases from different sources: time after which 50 or 100% of the glycosides were cleaved, *n* = 3

Hydrolysis	Recombinant rhamnosidase				Rhamnosidase from *A. niger*			
	37 °C		50 °C		37 °C		50 °C	
	50%	100%	50%	100%	50%	100%	50%	100%
NPRP	< 5 min	~ 20 min	< 5 min	~ 20 min	~ 10 h	~ 48 h	~ 6 h	~ 48 h
Rutin	20 min	130 min	15 min	170 min	~ 35 h	–	~ 22 h	–
Naringin	–	–	–	–	< 6 h	< 6 h	< 6 h	< 6 h
Hesperidin	90 min	–	70 min	–	~ 10 h	–	< 6 h	–

Rhamnosidase from *A. niger* is capable of hydrolysing also naringin to naringenin-7-*O*-glucoside. Using this enzyme, results in the most efficient cleavage of hesperidin followed by naringin and rutin (Table 1).

### Rhamnosidase and β-glucosidase activity of probiotics

**Fig. 1:**
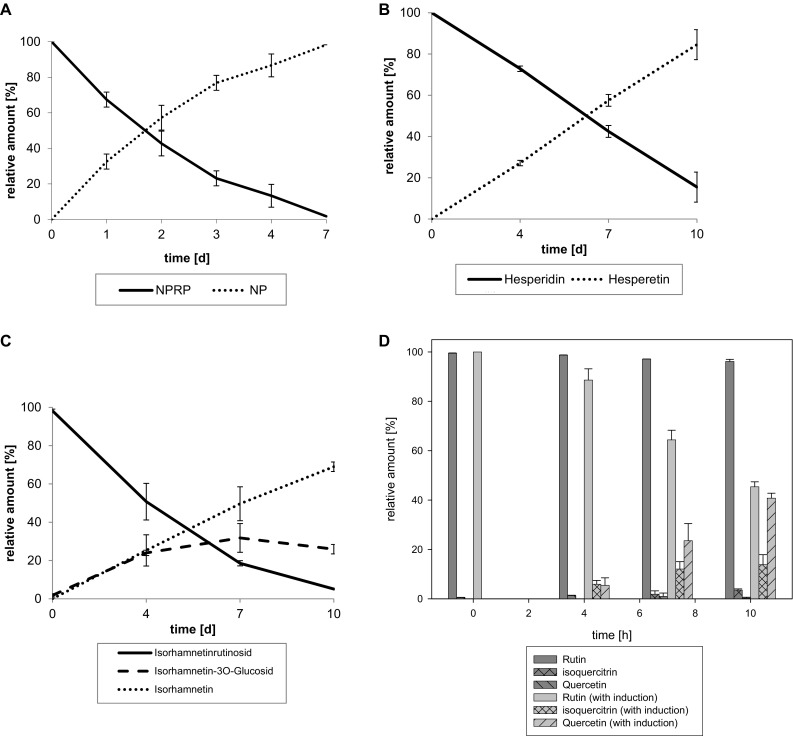
Rhamnosidase activity of *L. acidophilus* (as example). Deglycosylation of **A** NPRP, **B** hesperidin, **C** narcissin and **D** rutin without and with induction by narcissin in an extract of *C. johnstonii*

All probiotic strains exerted a significant rhamnosidase activity as indicated using NPRP as a substrate leading to more than 40% hydrolysis for 8 out of 14 strains after 1 day under anaerobic conditions (Table 2; Fig. 1A). In general, the hydrolysis rate of NPRP ranged between 20% (*L. reuteri*) and 68% [*Lactococcus* (*Lc*.) *lactis*] after 1 day of incubation and reached a total of > 86% hydrolysis after 4 days with exception of *L. reuteri*. The highest rhamnosidase activity after 1 day was exerted by *Lc. lactis* followed by *L. fermentum* and *L. brevis*, followed by *L. rhamnosus* GG, *B. longum* ssp. *infantis* and *L. paracasei* ssp. *paracasei* DSM 20312. The lowest rhamnosidase activity after 1 day was exerted by *L. reuteri*, followed by *L. delbrueckii* ssp. *bulgaricus*, and *S. salivarius* ssp. *thermophilus*.

**Table 2:** Hydrolysis of rhamnose (bioconversion) from NPRP, rutin, naringin and hesperidin or hydrolysis of glucose from NPGP at certain time points. The table lists the bioconversion in %

Incubation	Bioconversion (%)							
	NPRP		NPGP	Rutin	Naringin	Hesperidin		
	1 day	4 days	4 h	10 days	10 days	4 days	7 days	10 days
*B. animalis* ssp. *lactis* BB12	48 ± 0	88 ± 7	96	0	0	23 ± 3	52 ± 4	69 ± 9
*B. longum* ssp. *infantis*	50 ± 0	95 ± 3	100	0	0	17 ± 3	52 ± 11	54 ± 8
*L. acidophilus* LA-5	33 ± 4	87 ± 6	100	3 ± 0	0	27 ± 1	58 ± 3	84 ± 7
*L. paracasei* ssp. *paracasei* DN114001	43 ± 0	88 ± 5	100	0	0	8 ± 3	15 ± 4	25 ± 1
*L. paracasei* ssp. *paracasei* DSM 20312	49 ± 0	92 ± 8	100	0	0	20 ± 3	36 ± 8	56 ± 4
*L. paracasei* ssp. *paracasei* CRL 431	33 ± 9	90 ± 6	97	0	0	13 ± 2	34 ± 4	55 ± 11
*L. reuteri*	20 ± 6	74 ± 15	100	0	0	3 ± 1	12 ± 7	17 ± 7
*L. rhamnosus* GG	52 ± 8	97 ± 3	28	0	0	6 ± 1	8 ± 5	18 ± 5
*L. plantarum*	32 ± 4	98 ± 1	100	5 ± 0	0	11 ± 6	46 ± 6	70 ± 8
*L. brevis*	63 ± 5	100 ± 0	100	0	0	25 ± 3	53 ± 4	80 ± 5
*L. delbrueckii* ssp. *bulgaricus*	25 ± 9	98 ± 0	100	2 ± 0	0	21 ± 9	34 ± 8	48 ± 8
*Lc. lactis* ssp. *lactis*	68 ± 0	100 ± 0	95	0	0	15 ± 2	39 ± 5	57 ± 1
*L. fermentum*	63 ± 2	100 ± 0	94	0	0	21 ± 1	43 ± 1	66 ± 5
*S. salivarius* ssp. *thermophilus*	26 ± 3	86 ± 6	100	4 ± 0	0	18 ± 5	43 ± 6	55 ± 5

This is defined of the percentages of rhamnose cleaved from the glycosides, *n* = 3

The β-glucosidase activity is higher than the rhamnosidase activity. In fact, NPGP is converted into the aglycone to an extent of 94–100% already after 4 h (except *L. rhamnosus* GG) and totally converted after 1 day by all 14 included strains (Table 2; supplements).

### Hydrolysis of rutin, hesperidin and naringin by probiotics

The rate of hydrolysis of rhamnose from flavonoid rhamnoglucosides was highly dependent on the aglycon (Table 2). After cleavage of the rhamnose, the glucose is cleaved rapidly.

Rutin was hardly hydrolysed by probiotic rhamnosidases with a conversion (to isoquercitrin, following by hydrolysis to quercetin) ranging from 0 to 2% after 4 days, 0–3% after 7 days and 0–5% after 10 days by four strains, namely *L. plantarum, L. delbrueckii* ssp. *bulgaricus, S. salivarius* ssp. *thermophilus* and *L. acidophilus*.

The conversion rate of hesperidin was significantly higher ranging from 8% (*L. paracasei* DN114001) to 27% (*L. acidophilus*) after 4 days, 9% (*L. rhamnosus* GG) up to 54% (*L. brevis*) after 7 days and 25 up to 80% (*L. paracasei* DN114001) after 10 days (*L. brevis*) (Table 2). Hesperidin was hydrolyzed most efficiently by *L. acidophilus*, followed by *L. brevis* and *B. animalis* ssp. *lactis*. The hydrolysis of hesperidin was least efficient by *L. reuteri* and *L. rhamnosus* GG. Since the hydrolysis of the glucose after the hydrolysis of the rhamnose is much faster, only the aglycone hesperetin but not the hesperetin-7-*O*-glucoside can be found (Fig. 1B, supplements).

Naringin was not hydrolysed by any of the used probiotic bacteria.

### Cleavage of rhamnoglucosides from *C. johnstonii* extract by probiotics

The extract of *C. johnstonii* contains rutin and narcissin as main components. Narcissin is converted significantly faster as rutin or hesperidin. In fact, narcissin is converted by 14% (*L. reuteri*) to 56% (*L. acidophilus*) after 4 days, by 15% (*L. reuteri*) to 87% (*L. fermentum*) after 7 days and to more than 80% by ten strains after 10 days (Table 3). The glucoside (isorhamnetin-3-glucoside) and aglycone (isorhamnetin) can be detected in parallel. As expected, the relative amount of aglycone to glucoside increases with the duration of incubation (Fig. 1C, supplements).

**Table 3:** Hydrolysis of rhamnose (bioconversion) from narcissin and rutin in *C. johnstonii* extract at certain time points; percentages of cleaved glycosides. n = 3

	Bioconversion (%)					
	Narcissin			Rutin		
	4 days	7 days	10 days	4 days	7 days	10 days
*B. animalis* ssp. *lactis*	36 ± 6	72 ± 1	93 ± 10	3 ± 2	28 ± 1	57 ± 1
*B. longum* ssp. *infantis*	47 ± 1	85 ± 1	84 ± 13	15 ± 1	45 ± 2	73 ± 3
*L. acidophilus*	56 ± 1	77 ± 4	86 ± 6	11 ± 4	36 ± 4	55 ± 2
*L. paracasei* DN114001	22 ± 2	67 ± 2	95 ± 1	3 ± 1	26 ± 2	66 ± 1
*L. paracasei* DSM 20312	33 ± 7	54 ± 15	89 ± 1	8 ± 1	21 ± 3	50 ± 3
*L. paracasei* CRL431	22 ± 1	38 ± 3	52 ± 4	2 ± 2	6 ± 2	18 ± 5
*L. reuteri*	14 ± 9	15 ± 7	25 ± 14	0	0	5 ± 2
*L. rhamnosus* GG	22 ± 8	32 ± 4	72 ± 3	3 ± 2	13 ± 5	38 ± 11
*L. plantarum*	56 ± 9	87 ± 5	94 ± 3	6 ± 0	16 ± 1	26 ± 2
*L. brevis*	40 ± 5	79 ± 7	88 ± 8	10 ± 2	38 ± 11	64 ± 11
*L. delbrueckii* ssp. *bulgaricus*	38 ± 4	47 ± 3	81 ± 15	4 ± 1	20 ± 6	32 ± 6
*Lc. lactis* ssp. *lactis*	31 ± 8	69 ± 7	98 ± 5	6 ± 1	45 ± 13	62 ± 14
*L. fermentum*	42 ± 3	87 ± 7	97 ± 1	12 ± 0	51 ± 13	78 ± 9
*S. salivarius* ssp. *thermophilus*	42 ± 7	60 ± 9	71 ± 10	7 ± 0	21 ± 4	33 ± 0

Interestingly, in the presence of narcissin, the rhamnosidase activity is induced, so that the conversion of rutin is enabled or enhanced (Table 3; Fig. 1D, supplements). In fact, rutin is converted by up to 15% after 4 days (*B. infantis*), up to 51% after 7 days (*L. fermentum*) and up to 78% after 10 days (*L. fermentum*). Rhamnosidase activity was induced most efficiently in *L. fermentum, B. longum* ssp. *infantis* and *Lc. lactis*. However, rhamnosidase was hardly induced in *L. reuteri*. The hydrolysis products isoquercitrin and quercetin can be found in parallel with increasing amounts of quercetin with longer incubation duration.

### Expression of rhamnosidase in probiotics

**Fig. 2:**
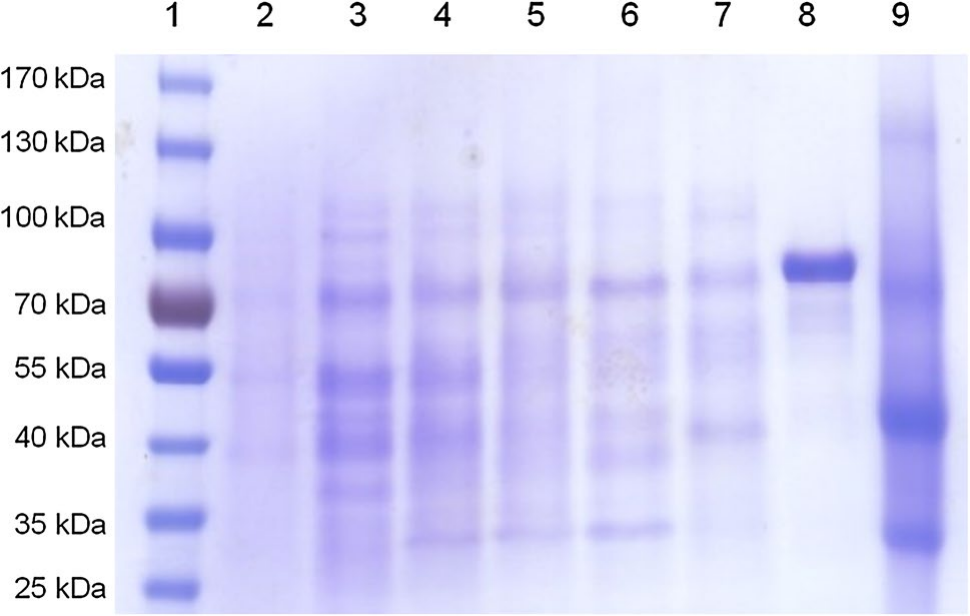
SDS-PAGE: (1) marker; (2) lysate of *L. acidophilus* without induction by NPRP; lysate of *L. acidophilus* with induction using NPRP after incubation for (3) 10 days, (4) 7 days, (5) 4 days, (6) one day, (7) without incubation; (8) recombinant rhamnosidase (20 mg/mL); (9) enzyme mixture from *A. niger* (cellulase; 75 mg/mL)

The expression of rhamnosidase is induced by the substrates as shown with NPRP in *L. acidophilus* (Fig. 2). It could be shown that the amount of rhamnosidase increases with the incubation time. The probiotic rhamnosidase was found to have a molecular weight of 80–90 kDa which is similar to the recombinant rhamnosidase with 90 kDa. The rhamnosidase in the enzyme mixture from *A. niger* had 80 kDa.

## Discussion

Due to their occurrence as glycosides, the bioavailability and bioactivity of polyphenols are limited, especially for rhamnoglucosides which cannot be cleaved by human intestinal enzymes. A few studies reported the release of rhamnose from flavonoid rhamnoglucoside by gut bacteria [23]. However, information on the rhamnosidase activity of probiotics is still limited. Previous studies have shown the occurrence and the properties of rhamnosidases from *L. plantarum, L. acidophilus* [24, 25] and *Pediococcus acidilactici* [26]. Another study has shown the hydrolysis of hesperidin by 6 out of 33 bifidobacteria strains within 48 h [6]. The results from our study provide further evidence of rhamnosidase activity in probiotics and of its substrate specificity. Rhamnosidase activity was found in all strains as shown with NPRP as substrate although with some differences of the activity among the strains. However, naringin could not be cleaved by any strain; rutin was cleaved by four strains to an extent below 5%, whereas the hydrolysis of narcissin and hesperidin was more rapid and to a higher extent. The preferred cleavage of hesperidin in contrast to rutin is consistent with the results from Amaretti et al. [6]. The preference for cleaving hesperidin (1–6 bonds) and no activity on naringin (1–2 bonds) conforms to previous studies with *Pediococcus acidilactici* or *L. plantarum* [25, 26]. In contrast to these and our results, Bang et al. [27] isolated a bifidobacterium (*B. dentium*) from human faeces samples with the ability to hydrolyse also rhamnose from the neohesperidose in naringin. Interestingly, this strain also was more effective in hydrolysing (1–6) bonds than (1–2) linkages of rhamnoglycosides.

In case of hesperidin no glucoside was detected, only the aglycone was found so the cleavage may be at least partly caused by rutinosidase. So far only fungal rutinosidase is known, so the contribution of bacterial rutinosidase has to be confirmed in further studies.

When comparing the results of Amaretti et al. [6] and our study, the conclusion can be drawn that lactobacilli are more effective in the cleavage of rhamnose from rutin and hesperidin than bifidobacteria, at least those used by Amaretti et al. [6]. A high rhamnosidase activity correlates well with the ability of cleaving hesperidin as shown for *L. brevis, Lc lactis* ssp. *lactis* and *L. fermentum*. A low rhamnosidase activity mostly correlates with a low hydrolysis rate of hesperidin as seen for *L. reuteri*. Interestingly, *L. rhamnosus* GG has a high rhamnosidase activity but low hydrolysis rate of hesperidin, indicating that the substrate specificity of the rhamnosidase enzyme from different probiotics differs. In strains with a high rhamnosidase activity as determined using NPRP, the probability for an induction of the rhamnose expression by narcissin and a following hydrolysis of rutin is increased. This was shown for *Lc. lactis, L. fermentum* or *B. longum* ssp. *infantis*. For *L. reuteri*, a low rhamnosidase activity (as shown with NPRP) correlates with a very low induction of the rhamnosidase using narcissin and a low following cleavage of rutin. Again, for *L. rhamnosus* GG no high induction of the rhamnosidase activity using narcissin was found even if the rhamnosidase activity using NPRP was high.

Here, the recombinant α-l-rhamnosidase was active towards hesperidin consistent to [28] and rutin, both of them show α-1,6 linkages to the β-d-glucosides. However, the enzyme was not active towards naringin, in which the L-rhamnose is α-1,2 linked to the β-d-glucoside. Interestingly, naringin is cleaved by rhamnosidase from *A. niger*. This goes conform with previous studies showing rhamnosidases from fungi to hydrolyse naringin [29]. The molecular weight of the rhamnosidase from *A. niger* and the probiotics or the recombinant one are different, potentially indicating a distinct glycosylation pattern which may lead to different substrate specificities.

Hydrolysis of the rhamnose from the main compounds (rutin, narcissin) of a *C. johnstonii* extract may increase bioactivity and bioavailability. The hydrolysis of narcissin by probiotics is efficiently and interestingly induces the activity of probiotic rhamnosidase so that rutin is also cleaved by all strains. Narcissin may be used as inducers for other flavonoid rhamnoglucosides as well. An induction of rhamnosidase activity by glycosides containing l-rhamnosyl moieties was suggested previously and shown for rutin by gut bacteria [25, 30]. In contrast to these previous studies, one easily hydrolysed compound of a plant extract helps to induce the cleavage of another compound in this extract. In such a way, one compound in the extract increases the bioavailability of another compound in this extract having a synergistic effect.

Considering the transit time of food in the gastro-intestinal tract, the velocity of hydrolysis by gut bacteria in the proximal as well as distal colon is relevant for hesperidin and narcissin. A pre-incubation of a flavonol glycoside containing supplement with bacteria may increase the yield of hydrolysis and thus bioavailability tremendously.

## Conclusion

14 probiotics hydrolysed hesperidin and narcissin, four probiotics hydrolysed a small amount of rutin, but no strain was able to cleave naringin. The efficiency of hydrolysis and substrate specificity was strain dependent. Recombinant α-l-rhamnosidase cleaved all rhamnoglucosides except naringin which was only cleaved by rhamnosidase from *A. niger*. One of the main compounds from *C. johnstonii* extract, narcissin, was not only hydrolysed by probiotic rhamnosidase, but induced rhamnosidase activity for cleavage of rutin. These findings provide further evidence of rhamnosidase activity in probiotics, making them to attractive sources for combined products with plant extracts with increased bioavailability of the active compounds.

## Electronic supplementary material

Below is the link to the electronic supplementary material.

Supplementary material 1 (DOCX 220 KB)
