# Supplementary material for: Rhamnosidase activity of selected probiotics and their ability to hydrolyse flavonoid rhamnoglucosides
Source: Bioprocess Biosyst Eng. 2017 Nov 10;41(2):221–8. doi: 10.1007/s00449-017-1860-5 (PMC5773629; doi:10.1007/s00449-017-1860-5)
Supplement: Supplementary file 1 — Supplementary material 1 (DOCX 220 KB) [file 449_2017_1860_MOESM1_ESM.docx]

**Supplementary material**

**Figure 1:** Deglycosylation of NPRP by different probiotics: (A) *B. animalis ssp. lactis*, (B) *B. infantis*, (C) *L. acidophilus*, (D) *L. paracasei* DN114001, (E) *L. paracasei* DSM 20312, (F) *L. paracasei* CRL431, (G) *L. reuteri*, (H) *L. rhamnosus* GG, (I) *L. plantarum*, (J) *L. brevis*, (K) *L. delbrückii ssp. bulgaricus*, (L) *Lc. lactis*, (M) *L. fermentum*, (N) *S. salivarius ssp. thermophilus*.

**Figure 2:** Deglycosylation of hesperidin by different probiotics: (A) *B. animalis ssp. lactis*, (B) *B. infantis*, (C) *L. acidophilus*, (D) *L. paracasei* DN114001, (E) *L. paracasei* DSM 20312, (F) *L. paracasei* CRL431, (G) *L. reuteri*, (H) *L. rhamnosus* GG, (I) *L. plantarum*, (J) *L. brevis*, (K) *L. delbrückii ssp. bulgaricus*, (L) *Lc. lactis*, (M) *L. fermentum*, (N) *S. salivarius ssp. thermophilus*.

**Figure 3:** Deglycosylation of narcissin by different probiotics: (A) *B. animalis ssp. lactis*, (B) *B. longum ssp. infantis*, (C) *L. acidophilus*, (D) *L. paracasei* DN114001, (E) *L. paracasei* DSM 20312, (F) *L. paracasei* CRL431, (G) *L. reuteri*, (H) *L. rhamnosus* GG, (I) *L. plantarum*, (J) *L. brevis*, (K) *L. delbrückii ssp. bulgaricus*, (L) *Lc. lactis*, (M) *L. fermentum*, (N) *S. salivarius ssp. thermophilus*.

**Figure 4:** Hydrolysis of rutin without and with induction by narcissin in an extract of *C. johnstonii*. (A) *B. animalis ssp. lactis*, (B) *B. longum ssp. infantis*, (C) *L. acidophilus*, (D) *L. paracasei* DN114001, (E) *L. paracasei* DSM 20312, (F) *L. paracasei* CRL431, (G) *L. reuteri*, (H) *L. rhamnosus* GG, (I) *L. plantarum*, (J) *L. brevis*, (K) *L. delbrückii ssp. bulgaricus*, (L) *Lc. lactis*, (M) *L. fermentum*, (N) *S. salivarius ssp. thermophilus*.

**Figure 1:**

**(A)**

**(B)**

**(C)**

**(D)**

**(E)**

**(F)**

**(G)**

**(H)**

**(I)**

**(J)**

**(K)**

**(L)**

**(M)**

**(N)**

**Figure 2:**

**(A)**

**(B)**

**(C)**

**(D)**

**(E)**

**(F)**

**(G)**

**(H)**

**(I)**

**(J)**

**(K)**

**(L)**

**(M)**

**(N)**

**Figure 3:**

**(A)**

**(B)**

**(C)**

**(D)**

**(E)**

**(F)**

**(G)**

**(H)**

**(I)**

**(J)**

**(K)**

**(L)**

**(M)**

**(N)**

**Figure 4:**

**(A)**

**(B)**

**(C)**

**(D)**

**(E)**

**(F)**

**(G)**

**(H)**

**(I)**

**(J)**

**(K)**

**(L)**

**(M)**

**(N)**
